# Tangible document sharing: handing over paper documents across a videoconferencing display

**DOI:** 10.3389/frobt.2024.1303440

**Published:** 2024-04-05

**Authors:** Kazuaki Tanaka, Kentaro Oshiro, Naomi Yamashita, Hideyuki Nakanishi

**Affiliations:** ^1^ Faculty of Information and Human Sciences, Kyoto Institute of Technology, Kyoto, Japan; ^2^ Independent Researcher, Yokohama, Japan; ^3^ Graduate School of Informatics, Kyoto University, Kyoto, Japan; ^4^ Faculty of Informatics, Kindai University, Higashi-osaka, Japan

**Keywords:** telepresence, media space, telerobotics, paper documents, videoconferencing

## Abstract

Conventional techniques for sharing paper documents in teleconferencing tend to introduce two inconsistencies: 1) media inconsistency: a paper document is converted into a digital image on the remote site; 2) space inconsistency: a workspace deliberately inverts the partner’s handwriting to make a document easy to read. In this paper, we present a novel system that eliminates these inconsistencies. The media and space inconsistencies are resolved by reproducing a real paper document on a remote site and allowing a user to handover the paper document to a remote partner across a videoconferencing display. From a series of experiments, we found that reproducing a real paper document contributes to a higher sense of information sharing. We also found that handing over a document enhances a sense of space sharing, regardless of whether the document is digital or paper-based. These findings provide insights into designing systems for sharing paper documents across distances.

## 1 Introduction

In face-to-face meetings, people often use paper documents rather than digital documents because paper documents are easy to edit and distribute (Paul et al., 1992; Sellen and Harper, 1997; Harboe et al., 2015). To use a paper document in a regular video conference, meeting members need to scan the document and send it in advance. Furthermore, if a member wants to share his/her hand drawings on the document, he/she must present it to a camera for live video streaming or scan and send it again. Such a cumbersome and indirect interaction may decrease the sense of information sharing, which is the sense of sharing identical information with a remote partner.

**FIGURE 1:**
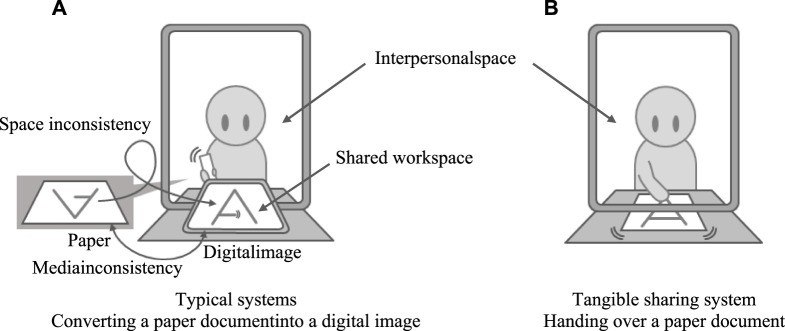
Systems for sharing paper documents across distance.

To share paper documents in remote collaboration more easily, many studies on media space have developed systems that simulate face-to-face collaboration in which users sit at the same desk and edit the same document (Ishii et al., 1993; Wellner, 1993; Robertson and Robinson, 1999; Takao et al., 2001; Paul et al., 2006; Izadi et al., 2007; Wilson et al., 2007). Figure 1A shows a typical system design. The system consists of a shared workspace and an interpersonal space. A shared workspace shows images of paper documents and users’ hands on the desk. An interpersonal space is a space where a remote partner’s upper body image is displayed. Although these systems successfully support tabletop collaboration across distances, they still introduce some inconsistencies into the workspace, which limits natural interaction among remote users.

In this paper, we focus on two inconsistencies that are commonly present in previous systems: 1) media inconsistency: many systems convert a paper document and a partner’s handwriting into a digital image. This conversion produces an inconsistency between the media used for showing the document on local and remote sites. 2) Space inconsistency: some systems deliberately turn the remote partner’s handwriting upside down to make the document easy to read for the remote partner. However, these systems introduce an inconsistency between the interpersonal space and shared workspace, which may deteriorate the users’ feeling of working in the same space.

The goal of our research is to see whether resolving these inconsistencies helps improve the users’ sense of sharing the same space. We believe that an increased sense of sharing the same space will lead to more natural interaction among remote users. To reach our goal, we designed a tangible sharing system, which is presented in Figure 1B. The system simulates a situation in which one person hands over a paper document to another who is sitting on the opposite side of the desk. Specifically, the system resolves the two inconsistencies by 1) reproducing a handwritten paper document in a remote space and 2) showing a remote user’s handover gesture in sync with the paper document so that it looks as if the remote user is handing over a document across the videoconferencing display.

By using our system, we investigate the following two research questions:

**Question 1: Effects of eliminating media inconsistency.** Converting a paper document into a digital document and using it instead of a paper document is an easy solution to resolve the media inconsistency problem. However, sharing digital documents may decrease the sense of sharing the same information with the remote partner because the remote partner’s handwriting cannot be observed in real time in conjunction with their hand movements. If a system can reproduce the remote partner’s handwritten document on real paper, the local partner may feel that it is indeed the remote partner’s handwritten paper document. As a result, the system may enhance the sense of sharing identical information with a remote partner, i.e., information sharing. In our experiment, we examine whether a user’s sense of information sharing is increased by reproducing a paper document compared with sharing a digital document.

**Question 2: Effects of eliminating space inconsistency.** Since systems that invert the handwriting of a remote partner introduce space inconsistency between the workspace and interpersonal space, it may decrease the sense of “space sharing,” which is the feeling of sharing the same space with a remote partner. On the other hand, such space inconsistency will not occur if a remote partner can handover a document to a user. As a result, the user may feel an increased sense of space sharing. In our experiment, we examine whether enabling a user to handover a document enhances the sense of space sharing compared with a system presenting an inverted digital image of a remote partner’s handwriting.

Our system that eliminates space inconsistency has the effect of conveying the remote partner’s body movements through document sharing, as well as expressing the spatial connections of workspaces. It has been reported that presenting the partner’s body movements via a physical embodiment, such as humanoid robots, increases the sense of space sharing (Tanaka et al., 2015). Our system presents the physical movement of the paper document in sync with the partner’s handover gesture, and so, it has the potential to enhance the sense of spatial sharing compared to the typical system. However, if the movement of the document is not synchronized with the handover gesture (e.g., it moves automatically), it may be less effective in enhancing the sense of space sharing. We, therefore, examine whether the movement of a document in sync with the handover gesture enhances the sense of spatial sharing.

In Experiment 1, we confirmed the superiority of our system over the typical systems in the sense of information and space sharing. In Experiment 2, we examined the effectiveness of our system in more detail by dividing it into two factors: the media factor (paper/digital) and the gesture factor (with/without handover gesture). In Experiment 3, we verified the difference in effectiveness when a user hands over and receives a document via our system.

## 2 Related works

### 2.1 Media space

Due to the convenience of paper documents, previous studies on media space have designed many systems for sharing paper documents across distances (Ishii et al., 1993; Wellner, 1993; Robertson and Robinson, 1999; Takao et al., 2001; Everitt et al., 2003; Paul et al., 2006; Izadi et al., 2007; Wilson et al., 2007). For example, Agora (Paul et al., 2006) projected an image of a remote partner’s workspace on a local workspace and a life-size upper body image on a vertical screen that stands across the desk. Most systems project a partner’s workspace onto a local desk (Wellner, 1993; Robertson and Robinson, 1999; Takao et al., 2001; Paul et al., 2006; Wilson et al., 2007) or present it on a tabletop display (Ishii et al., 1993; Izadi et al., 2007) to share paper documents. In such systems, the media inconsistency problem occurs since a paper document on the workspace is presented as a digital image in a remote workspace. Furthermore, the space inconsistency also occurs because the workspace image is flipped to make the document easier to read.

Another study developed a system that shares physical post-it notes on a board over long distances (Everitt et al., 2003). In this system, a post-it note put on a local board is presented as a digital image on a remote board. Therefore, there is a media inconsistency problem. The board also shows an image of a remote partner’s shadow to present the partner’s pointing gesture. This system may resolve the space inconsistency problem. However, there is another inconsistency problem in that the partner does not exist in front of the shadow. This is known as the physical inconsistency problem and has been shown to decrease the sense of space sharing (Nakanishi et al., 2017). Similarly, for systems which do not have an interpersonal space (Wellner, 1993; Takao et al., 2001; Wilson et al., 2007), physical inconsistency occurs because the partner cannot be seen even though a workspace is shared with the partner.

Previous studies have also designed systems for sharing a digital workspace (Tang and Minneman, 1990; Gaver et al., 1991; Ishii and Kobayashi, 1992; Tuddenham and Robinson, 2009; Judge et al., 2011; Weibel et al., 2011). For instance, VideoDraw (Tang and Minneman, 1990) shares handwritten notes on a display screen to simulate a situation where users are writing and drawing on the same pad of paper. Such systems resolved the media inconsistency problem by sharing digital documents instead of real paper documents. However, the space inconsistency problem exists due to the inverted workspace. Other systems share documents and users’ handwriting on a digital whiteboard (Elrod et al., 1992; Kunz et al., 2010). These systems have the same inconsistency problem as the system for sharing post-it notes, i.e., the physical inconsistency problem.

Unlike these previous systems, our tangible sharing system eliminates both the media and space inconsistency problems by allowing a user to handover a paper document to a remote partner. The next section will describe the configuration of our system.

### 2.2 Senses of information sharing and space sharing

Many systems present a remote partner’s hand image (Tang and Minneman, 1990; Wellner, 1993; Takao et al., 2001; Paul et al., 2006; Izadi et al., 2007; Wilson et al., 2007; Weibel et al., 2011) or shadow (Tuddenham and Robinson, 2009) on a shared workspace. In such systems, the sense of information sharing may increase because the partner’s hand image will improve the certainty that the partner is actually writing. However, presenting hand images will decrease the sense of space sharing since it further emphasizes space inconsistency.

There are some previous systems that resolve the space inconsistency problem. ClearBoard (Ishii and Kobayashi, 1992) simulates a situation where users communicate through a glass board; the users can see documents on the board and handwrite on it. However, displaying information on the glass board emphasizes the boundary surface between remote and local sites more, which may reduce the sense of space sharing.

Lazy Susan (Wesugi and Miwa, 2006) simulates a situation where users share objects across the same turntable. The rotations of local and remote turntables are synchronized. In this system, remote documents are projected on a local turntable; hence, the sense of information sharing will decrease due to media inconsistency.

This Lazy Susan system has been adopted into mirror-type videoconferencing (Nakanishi et al., 2017). In mirror-type videoconferencing, a display works as a mirror so that it shows both local and remote users. Mirror-type videoconferencing has the inconsistency that the remote partner does not exist in the local space. The study has resolved this inconsistency by creating a partition that blocks a local user from seeing the presence or absence of the partner. Additionally, sharing a physical object through the Lazy Susan system facilitated a user’s imagination that the partner exists behind the partition. Compared with mirror-type videoconferencing, a display of normal videoconferencing works as a window. In our system, handing over a paper document could facilitate the subjects’ imagination that the partner is sitting on the other side of a window.

## 3 Design

**FIGURE 2:**
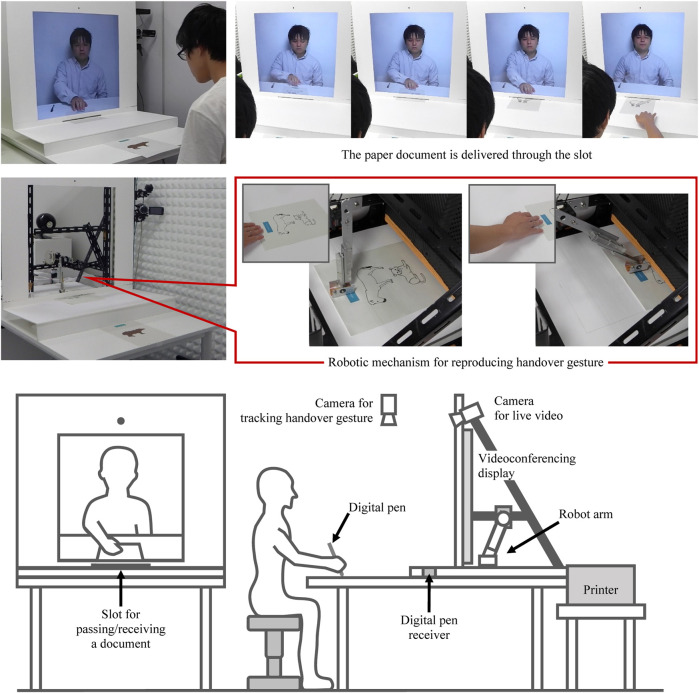
Configuration of our tangible sharing system that enables the user to handover a paper document.

Figure 2 shows the configuration of our tangible sharing system. Similar to previous systems, our system consists of a workspace and an interpersonal space. To form an interpersonal space, a vertical videoconferencing display is located on the desk. A workspace on the desk is equipped with a digital pen for obtaining users’ handwriting or drawing on a paper document.

When a user draws on a paper document, the digital pen will obtain the hand-drawn image. Once the user finishes drawing, he puts a cap on the digital pen to turn it off. Then, the user’s hand-drawn image is automatically sent to the remote site and printed by a printer. When the user decides to share it with the remote partner, he can pass the paper document through a slot located at the intersection of the videoconferencing display and the workspace. A camera for live video streaming is mounted on the videoconferencing display, and a camera for tracking handover gestures is mounted above the user. Behind the videoconferencing display, the printer and a robot arm are installed. When the user pushes the paper document into the slot, the camera recognizes his hand movement. Then, at the remote site, the robot arm pushes the printed document into the slot so that its movement synchronizes with the user’s hand movement. In this way, the system can provide the illusion that the user is handing over a paper document across the videoconferencing display.

It takes time after the user finishes drawing until a paper document is replicated at a remote site. Most of the time is spent printing a paper document on the printer. In the experiments described below, the following method solved this printing time problem. In Experiments 1 and 2, the experimenter’s handwriting was printed out in advance and prepared for the subject site. Thus, the experimenter was able to immediately handover the paper document to the subjects. In contrast, in Experiment 3, the subjects’ hand-drawn image had to be printed out on the experimenter’s site. Therefore, in order to buy time for printing, the experimenter instructed the subject to handover the paper after having a 1-min conversation. In order to put our system into practical use, it is necessary to have a conversation until the printing process is completed, as demonstrated in Experiment 3, or to use technology that prints paper at high speed.

## 4 Data collection

A total of 64 undergraduate students participated as subjects in the three experiments (Experiment 1: 9 females and 9 males; Experiment 2: 17 females and 19 males; and Experiment 3: 3 females and 7 males).

### 4.1 Survey

In our experiments, the subjects answered the survey questions to evaluate the sense of information sharing and space sharing. They were answered on a 7-point Likert scale: 1: strongly disagree, 4: neutral, and 7: strongly agree.

To evaluate the sense of information sharing, defined as the sense of sharing identical information with a remote partner, we asked the following survey question. This item is intended to measure the degree to which the subjects feel that information (i.e., the partner’s handwriting) is directly conveyed to them without system intervention when received from their conversation partner. We expected that receiving the handwriting on a paper document rather than on a digital document would enhance this feeling.

• I felt that the contents of the conversation partner’s handwriting were conveyed.

The sense of space sharing is a concept similar to social presence (De Greef and Ijsselsteijn, 2001; Pereira et al., 2014) or co-presence (Hoppe et al., 2020) in a broad sense. Social presence is defined as the “sense of being together with another (Biocca et al., 2001).” Since this concept also applies to interaction with autonomous agents and robots (Pereira et al., 2014), it is sometimes referred to as social telepresence (Tanaka et al., 2015; Nakanishi et al., 2017) and the sense of space sharing (Tanaka et al., 2021) in studies on remote communication. When evaluating this feeling in remote communication, it has been found that the expression “being with the conversation partner in the same room” is effective (Tanaka et al., 2015; Nakanishi et al., 2017; Tanaka et al., 2021). We, therefore, asked the following survey question to evaluate the sense of space sharing.

• I felt as if I were talking with my conversation partner in the same room.

For these items, subjects wrote the reasons for their answers in the free text field. Common opinions were extracted from these free descriptions and utilized in the discussion. We confirmed through interviews with the subjects that they interpreted and answered these items as we intended.

### 4.2 Video recordings

The experiments were videotaped, and we logged the subjects’ activities throughout the experiments. The video data were used for analyzing how the subjects interacted with the system. More specifically, we observed the subjects’ behavior to explore how resolving media and space inconsistencies affected their interaction with the paper document as well as with the experimenter. We focused on the subjects’ gaze movement because gaze movement often reflects people’s level of confidence. For example, an unsteady gaze movement reflects low confidence in the user (Dewen et al., 2022). In our experiment, the subjects often shifted their gaze between the document and the experimenter after the experimenter shared the document with the subjects. Thus, we focused on the period after the experimenter shared his drawing on the document and started talking. We calculated the frequency of gaze movement by counting the number of each subject’s gaze movements and dividing it by the conversation time during that time period. The conversation time was approximately 40 s.

It is known that in face-to-face conversations, people basically look at their partner’s face (Turkstra, 2005; Freeth et al., 2013; Vabalas and Freeth, 2016), and the same is true when talking with a video-taped partner (Freeth et al., 2013). However, when there is an object (i.e., a paper document) to direct their gaze to other than the partner, it is unclear how people direct their gaze toward the partner or the object during a conversation. In video calls, it is difficult to see where the remote partner is looking (Gale and Monk, 2000), so a previous study tried to make it easier to see which document or person the remote partner is looking at in the local space during a multi-person video call (Le et al., 2019). In this study, we examined how people direct their gaze toward a document on a workspace and a partner’s video during 

## 5 Experiment 1

As described above, our system eliminates media and space inconsistency problems by enabling users to handover a paper document to a remote partner. A system that shares a digital document instead of a paper document can also eliminate the media inconsistency problem, but the space inconsistency problem remains unsolved due to the inverted image. In the first experiment, we investigated which system effectively resolves the media inconsistency problem by enhancing the sense of information sharing Furthermore, we also investigated whether eliminating the space inconsistency problem enhanced these sensations. For these purposes, we compared these systems with normal videoconferencing.

### 5.1 Procedure

Before conducting the experiment, an instructor told the subjects that their conversation partner (the experimenter) was in another room. On the experimenter’s desk, a picture of an animal without its fur pattern was placed in advance. Then, the experimenter drew the fur pattern on the picture and passed it to the subject in accordance with the method of the experimental condition. When the subject received the picture, the experimenter conducted a quiz, asking the subject for the reasons for the fur pattern. Finally, the experimenter said goodbye and ended the conversation. This series of procedures was repeated under all three conditions. After experiencing the three conditions, the subjects answered a questionnaire to evaluate their sense of information sharing and space sharing.

### 5.2 Conditions

**FIGURE 3:**
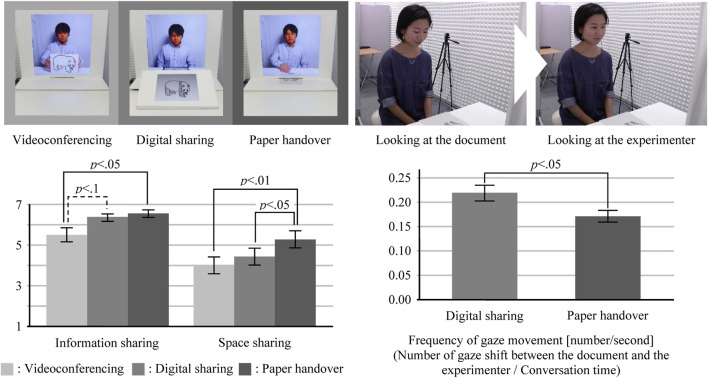
Results of Experiment 1 (upper left: conditions; lower left: questionnaire result; upper right: example of the subject’s gaze movement; and lower right: frequency of gaze movement).

The systems used in the three conditions are shown in Figure 3 (upper left). Experiment 1 was a within-subject design where each subject experienced all three conditions, whose order was counterbalanced. Each condition was assigned a different conversation topic, i.e., animal pictures. The combination of conditions and topics was also counterbalanced. We set the three conditions as follows:

**Paper handover condition (resolving both media and space inconsistencies):** in this condition, the experimenter hands over his handwritten paper document to a subject through our system. Media inconsistency is resolved by reproducing a paper document in a remote space, and space inconsistency is resolved by enabling the handover gesture of the paper document.

**Digital sharing condition (resolving media inconsistency only):** in this condition, a document is presented digitally on two touch-screen displays: one on the experimenter’s table and the other on the subject’s table. When an experimenter writes on the touch-screen display, the writing also appears on the subject’s touch-screen display in real time. Media inconsistency is resolved by both users sharing a digital document/image. However, space inconsistency exists because the digital documents are shown in a manner where remote users can view them in the same direction.

**Videoconferencing condition (baseline condition):** in this condition, users only had a videoconferencing display for presenting their upper body images. The experimenter showed his handwritten paper document to the subject by lifting up the paper and showing it to the camera mounted on the video-conferencing display. This condition does not deal with media and space inconsistency problems because there is no workspace available to share a document. Meanwhile, this condition serves as a baseline to see whether the workspace with/without media and space inconsistency enhances the senses of information sharing and space sharing.

### 5.3 Results

To test the effects of resolving media and space inconsistencies, we conducted a one-way repeated-measures analysis of variance (ANOVA), followed by a Bonferroni correction for the questionnaire result. Figure 3 (lower left) shows the results of the comparison. Each box represents the mean values, and each bar represents the standard error of the mean.

There was a significant main effect on the sense of information sharing (*F* (2, 34) = 6.081; *p* < 01). Multiple comparisons showed that the paper handover condition had a higher sense of information sharing than videoconferencing (*p* < .05). In addition, the digital sharing condition tended to have a higher sense of information sharing than the videoconferencing condition (*p* = .093). However, the difference between the paper handover and digital sharing conditions was not significant. These results mean that the paper handover condition enhanced the sense of information sharing. However, the effect of the digital sharing condition on this sensation was weak.

There was a significant main effect on the sense of space sharing (*F* (2, 34) = 8.926; *p* < 01). Multiple comparisons showed that the paper handover condition had a higher sense of space sharing than the videoconferencing (*p* < 01) and digital sharing (*p* < 05) conditions. This result means that the paper handover condition enhanced the sense of space sharing.

From an analysis of the video recordings, we found that most subjects were basically looking at a document but sometimes looked at the experimenter, e.g., when answering the experimenter’s question or when something was unclear. We conducted this analysis for 16 of 18 subjects due to the failure to record two subjects’ faces. As shown in Figure 3 (lower right), a paired *t*-test showed that the frequency of such gaze movement was significantly higher in the digital sharing condition than that in the paper handover condition (*t* (15) = 2.866, *p* < 05).

Overall, our results show that handing over a paper document was successful in eliminating media and space inconsistencies. Resolving the inconsistency problems was effective in enhancing the senses of information sharing and space sharing. Furthermore, the subjects’ gaze movements were more stable in the paper handover condition. These results are consistent in that they all show that subjects felt more familiar with the environment and had a better understanding of what was going on. However, the results did not show which factor contributed to these effects: reproducing a paper document or a handover gesture. The second experiment examined these factors.

## 6 Experiment 2

We assumed that reproducing a paper document enhances the sense of information sharing and handing it over enhances the sense of space sharing. To examine these assumptions, this experiment investigates two factors: 1) media factor: whether a document is represented in a digital image or on real paper and 2) gesture factor: whether a movement of a document is synchronized with a remote partner’s handover gesture. This experiment used the same procedure as Experiment 1.

### 6.1 Conditions

**FIGURE 4:**
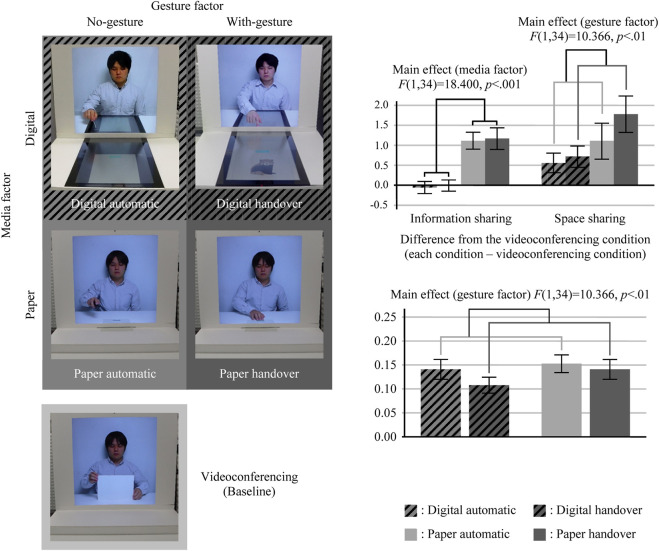
Results of Experiment 2 (left: conditions; upper right: questionnaire result; and lower right: frequency of gaze movement).

The systems used in the five conditions are shown in Figure 4 (left). We divided these conditions into two experiments because experiencing all five experimental conditions would have been a heavy burden for the subjects. Experiment 2-1 included videoconferencing, digital automatic, and digital handover conditions. Experiment 2-2 included videoconferencing, paper automatic, and paper handover conditions. The first 18 subjects (9 females and 9 males) and the other 18 subjects (8 females and 10 males) participated in Experiments 2-1 and 2-2, respectively. These experiments had a within-subject design. Therefore, the gesture factor was the within-subject factor, but the media factor was the between-subject factor. In these experiments, the order of the three conditions was counterbalanced. The combination of conditions and topics was also counterbalanced. We set the five conditions as follows:

**Paper handover condition (paper, with handover gesture):** this was the same as the paper handover condition in Experiment 1.

**Paper automatic condition (paper, no handover gesture):** the experimenter shared a paper document with a subject through our system, but he used a remote control to pass the paper. When he pushed the button on the remote control, the paper document automatically moved from the experimenter site to the subject site. Thus, the subject did not see the experimenter’s handover gesture.

**Digital handover condition (digital, with handover gesture):** The experimenter shared a digital document on a tabletop display (TTD). He used a handover gesture, i.e., swipe input, to pass the document. The subject could see the handover gesture synchronized with the movement of the on-screen digital document.

**Digital automatic condition (digital, no handover gesture):** The experimenter shared a digital document on the TTD. He used the touch input on the TTD to send the document. When he touched the button on the TTD, the paper document automatically moved from the experimenter site to the subject site. Thus, similar to the paper’s automatic condition, the subject did not see the experimenter’s handover gesture.

**Videoconferencing condition (baseline condition):** In addition to these four conditions, we set the videoconferencing condition as a baseline condition. This was the same as the videoconferencing condition in Experiment 1. We compared the difference between the evaluation values of the four conditions and the videoconferencing condition.

### 6.2 Results

We conducted a two-way mixed-measures ANOVA (the media factor: between and the gesture factor: within) for the questionnaire result. To conduct this analysis, in Experiments 2-1 and 2-2, we used the evaluation values of the automatic/handover conditions minus the evaluation values of the videoconferencing (baseline) condition. Figure 4 (upper right) shows the results of the ANOVA. Each box represents the mean values, and each bar represents the standard error of the mean.

There was a significant main effect of the media factor on the sense of information sharing (*F* (1, 34) = 18.400; *p* < 001) but not the gesture factor. This result means that reproducing a paper document enhanced the sense of information sharing, regardless of whether the experimenter’s handover gesture was presented or not.

There was a significant main effect of the gesture factor on the sense of space sharing (*F* (1, 34) = 10.366; *p* < 01) but not the media factor. This result means that presenting the experimenter’s handover gesture synchronized with the movement of the document enhanced the sense of space sharing, regardless of whether the document is digital or paper-based.

Figure 4 (lower right) shows the result of the frequency of gaze movement. A two-way mixed-measures ANOVA indicated that there was a significant main effect of the gesture factor (*F* (1, 34) = 5.133; *p* < 05). This means that the document movement accompanied by the handover gesture reduced the frequency.

The questionnaire results were almost as we expected, but one question arises: is passing or receiving a document more effective for enhancing the sense of space sharing? The third experiment addressed this question.

## 7 Experiment 3

The second experiment clarified that handing over a document contributes to enhancing the sense of space sharing. A document is handed over in a direction: passing/receiving. We assumed that the subjects would feel an increased sense of space sharing when they passed a document. When they receive a document, they can easily imagine that a pre-printed document was prepared at the subject site. In contrast, when they pass their handwritten paper document, preparing it in advance is difficult. We, therefore, considered that passing a handwritten paper document is more effective than receiving it. This experiment examined this assumption. In this experiment, the analysis of the frequency of gaze movement was not conducted since the places of a document after handing it over and receiving it were different.

### 7.1 Procedure

In each condition, the experimenter presented a stuffed animal and asked the subject to draw a picture of the animal on a paper document. Then, the experimenter told the subject facts about the animal. Finally, the experimenter said goodbye and ended the conversation. This series of procedures was repeated under three conditions. After experiencing the three conditions, the subjects answered a questionnaire to evaluate their sense of space sharing.

### 7.2 Conditions

**FIGURE 5:**
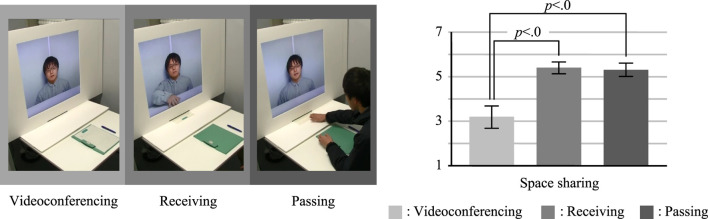
Conditions and results of experiment 3 (Left: Conditions, Right: Questionnaire result).

The systems used in the three conditions are shown in Figure 5 (left). This experiment was a within-subject design where each subject experienced all three conditions, whose order was counterbalanced. Each condition was assigned a different conversation topic, i.e., stuffed animals. The combination of conditions and topics was also counterbalanced. We set the three conditions as follows:

**Videoconferencing condition:** on the subject’s desk, paper for drawing a picture was placed in advance. The subject did not pass/receive the document.

**Receiving condition:** the subject received the paper from the experimenter. The subject did not pass it on to the experimenter.

**Passing condition:** on the subject’s desk, the paper was placed in advance. After the experimenter told the subject animal facts, the subject passed the paper to the experimenter.

### 7.3 Results

The one-way repeated measures ANOVA showed a significant main effect on the sense of space sharing (F (2, 18) = 13.573; *p* < 01). Figure 5 (right) shows the result of the Bonferroni *post hoc* tests. The passing and receiving conditions had significantly higher senses of space sharing than the videoconferencing condition (*p* < 01, both conditions). However, contrary to our assumption, there was no significant difference between the passing and receiving conditions. This means that handing over a paper document enhances the sense of space sharing, regardless of the direction.

## 8 Discussion

### 8.1 Effects of eliminating media inconsistency

The experiments showed that reproducing a paper document as a paper document is effective for eliminating the media inconsistency problem. As a result, the user has an increased sense of sharing the same information with a remote partner, i.e., information sharing. As the reason for the increased sense of information sharing, many subjects (Experiment 1: 11 of 18 subjects and Experiment 2-2: 9 of 18 subjects) mentioned that they were able to pick up the paper document and see it. This means that the tangibility of a paper document is useful for sharing information.

Another way to eliminate the media inconsistency problem is to share digital documents. This study tested the two systems for sharing digital documents: the digital sharing condition in Experiment 1 and the digital handover and digital automatic conditions in Experiment 2-1. The digital sharing condition tended to enhance the sense of information sharing, but the digital handover and digital automatic conditions did not. This indicates that presenting a partner’s handwriting process in real time also enhances the sensation. Four subjects in Experiment 1 mentioned the handwriting process as the reason for the increased sense of information sharing. In addition, two subjects who preferred both the paper handover and digital sharing conditions described that they actually saw what the experimenter was writing. From these subjects’ responses, it is considered that the certainty that the remote partner actually wrote on a document contributes to the sensation. Producing a real paper document and presenting a handwriting process are quite different approaches, but they may enhance the sensation by increasing this certainty.

The effect of producing a real paper document on the sense of space sharing was not significant in Experiment 2. As shown in Figure 4, the sense of space sharing seems higher in the paper conditions than in the digital conditions. However, seven subjects said that they felt a mechanical impression in the paper conditions since they noticed the noise of a servomotor. They also noted that especially the paper automatic condition seems like a facsimile. For this reason, the sense of space sharing in the paper conditions somewhat decreased. It is therefore thought that producing a real paper document may also enhance the sense of space sharing if our system is soundproofed enough so that the user cannot hear the noise of the servomotor.

### 8.2 Effects of eliminating space inconsistency

As we expected, eliminating the space inconsistency problem by handing over a document enhances the sense of space sharing, which is the feeling of sharing the same space with a remote partner. The movement of a document from a remote site to a local site across a videoconferencing display may facilitate the feeling that the local and remote spaces are continuously concatenated. However, the movement is effective only when synchronized with the remote partner’s handover gesture. As discussed in the previous section, moving the document with the remote control adversely affects the sense of space sharing due to giving a mechanical impression.

The movement of a document from a remote site to a local site was effective in reducing the frequency of gaze movement. In the digital sharing condition in Experiment 1, the subjects frequently shifted their gaze between the document and the experimenter to see where the experimenter was looking. In contrast, in the paper handover condition in Experiment 1 and all the conditions in Experiment 2, the subjects did not need to frequently check where the experimenter was looking because it was clear that there was no document on the experimenter’s site (after handing it over to the subjects).

The frequency of the digital sharing condition in Experiment 1 reaches 0.2, while the frequencies of the automatic conditions in Experiment 2 are approximately 0.15. The frequency was further reduced when the document movement was accompanied by the handover gesture. These results indicate the possibility that bringing media space closer to a face-to-face environment makes the users’ gaze movements more stable. This change in user behavior may be the reason why resolving the space inconsistency problem increases the sense of shared space.

We expected the sense of space sharing to be higher when the subjects pass a document than when they receive it because preparing the subjects’ handwriting on the remote site in advance seems more difficult. Contrary to this expectation, the direction of the document’s movement, pass/receive, did not affect the sensation. As the reason for the increased sense of space sharing, one subject mentioned that the experimenter received a document at the time when he handed it over. In addition, he also described the synchronization between the on-screen experimenter’s hand movement and the physical movement of a paper document. This result supports the effectiveness of presenting the synchronization between users’ handover gestures and a document’s movement in enhancing the sense of space sharing.

In addition, Experiment 2 showed that handing over a document is effective regardless of whether the document is represented as real paper or an on-screen digital image. Nevertheless, Figure 4 shows that the difference between the automatic and handover conditions seems more remarkable in the paper conditions than in the digital conditions. Seven subjects in Experiment 2-2 (comparing paper conditions) mentioned the synchronization between the movement of a document and the partner’s handover gesture, but only two subjects in Experiment 2-1 (comparing digital conditions) mentioned it. This may indicate a floor effect. If a higher-resolution touch display is used for the TTD, handing over digital documents may become more effective.

## 9 Conclusion

Conventional systems for sharing documents across distances often have two inconsistencies: 1) media inconsistency: a paper document is converted into a digital image on the remote side. Therefore, there is an inconsistency of media that represent the document between local and remote sides. 2) Space inconsistency: a workspace presents the remote partner’s handwriting, which is turned upside down to make the document easy to read. We assumed that eliminating these inconsistencies would enhance the senses of information sharing and space sharing. In this study, to eliminate these inconsistencies, we developed and tested a novel system that allows a user to handover a paper document to a remote partner across a videoconferencing display.

A series of experiments revealed the effects of eliminating space inconsistency on the sense of space sharing. When documents move across a videoconferencing display, the sense of space sharing increases regardless of whether the documents are digital or paper-based. However, the movement is effective only when synchronized with the partner’s handover gesture. The document’s movement with the handover gesture facilitates the feeling that the local and remote spaces are continuously concatenated. Furthermore, it is also effective for reducing movements of the user’s gaze to care about where the partner is looking. There is a possibility that our system makes users feel more familiar with the environment and gain a better understanding of what is happening. As a result of such improvements in user behavior, the feeling of space sharing may increase.

The series of experiments also revealed the effects of eliminating media inconsistency on the sense of information sharing. Reproducing a remote partner’s handwritten document on paper enhances the sense of information sharing, regardless of whether the document’s movement is synchronized with the handover gesture or not. Sharing documents through a system raises the suspicion that the system fakes a document in an extreme case. Compared with sharing a digital document, receiving a document as a paper document seems to increase the certainty that the remote partner actually wrote it. The sense of information sharing improves with this certainty.

The findings of this study contribute to two telepresence research fields: media space and telerobotics. The synergistic effects of the media space technology, sharing a document across distance, and the telerobotics technology, manipulating a physical object across distance, enhance the senses of information sharing and space sharing. We hope that this study promotes the integration of these research fields.

## Data Availability

The original contributions presented in the study are included in the article/Supplementary Material; further inquiries can be directed to the corresponding author.
